# A computational approach to identify point mutations associated with occult hepatitis B: significant mutations affect coding regions but not regulative elements of HBV

**DOI:** 10.1186/1743-422X-8-394

**Published:** 2011-08-08

**Authors:** Roberto Bruni, Mattia Prosperi, Cinzia Marcantonio, Alessandra Amadori, Umbertina Villano, Elena Tritarelli, Alessandra Lo Presti, Massimo Ciccozzi, Anna R Ciccaglione

**Affiliations:** 1 Department of Infectious, Parasitic and Immune-Mediated Diseases, Viral Hepatitis Section, Istituto Superiore di Sanità, Rome, Italy; 2 Department of Pathology, Immunology and Laboratory Medicine, Emerging Pathogens Institute, College of Medicine, University of Florida, Gainesville, Florida, USA; 3 Department of Infectious, Parasitic and Immune-Mediated Diseases, Epidemiology Section, Istituto Superiore di Sanità, Rome, Italy

**Keywords:** Hepatitis B Virus, occult infection, bioinformatics

## Abstract

**Background:**

Occult Hepatitis B Infection (OBI) is characterized by absence of serum HBsAg and persistence of HBV-DNA in liver tissue, with low to undetectable serum HBV-DNA. The mechanisms underlying OBI remain to be clarified. To evaluate if specific point mutations of HBV genome may be associated with OBI, we applied an approach based on bioinformatics analysis of complete genome HBV sequences. In addition, the feasibility of bioinformatics prediction models to classify HBV infections into OBI and non-OBI by molecular data was evaluated.

**Methods:**

41 OBI and 162 non-OBI complete genome sequences were retrieved from GenBank, aligned and subjected to univariable analysis including statistical evaluation. Their S coding region was analyzed for Stop codon mutations too, while S amino acid variability could be evaluated for genotype D only, due to the too small number of available complete genome OBI sequences from other genotypes.

Prediction models were derived by multivariable analysis using Logistic Regression, Rule Induction and Random Forest approaches, with extra-sample error estimation by Multiple ten-fold Cross-Validation (MCV). Models were compared by t-test on the Area Under the Receiver Operating Characteristic curve (AUC) distributions obtained from the MCV runs for each model against the best-performing model.

**Results:**

Variations in seven nucleotide positions were significantly associated with OBI, and occurred in 11 out of 41 OBI sequences (26.8%): likely, other mutations did not reach statistical significance due to the small size of OBI dataset. All variations affected at least one HBV coding region, but none of them mapped to regulative elements. All viral proteins, with the only exception of the X, were affected. Stop codons in the S, that might account for absence of serum HBsAg, were not significantly enriched in OBI sequences. In genotype D, amino acid variability in the S was higher in OBI than non-OBI, particularly in the immunodominant region. A Random Forest prediction model showed the best performance, but all models were not satisfactory in terms of specificity, due to the small sample size of OBIs; however results are promising in the perspective of a broader dataset of complete genome OBI sequences.

**Conclusions:**

Data suggest that point mutations rarely occur in regulative elements of HBV, if ever, and contribute to OBI by affecting different viral proteins, suggesting heterogeneous mechanisms may be responsible for OBI, including, at least in genotype D, an escape mutation mechanism due to imperfect immune control. It appears possible to derive prediction models based on molecular data when a larger set of complete genome OBI sequences will become available.

## Background

Hepatitis B virus (HBV) acute infection, often asymptomatic, can be followed by recovery or by virus persistence. At least three distinct clinical states of viral persistence have been defined based on serological findings: chronic hepatitis B, silent or "healthy" carrier, and "occult hepatitis B" [1].

In a typical persistent HBV infection, individuals are positive for both HBV DNA and HBsAg. In occult hepatitis B infection (OBI) individuals show very low level serum HBV DNA (< 200 IU/ml) but are negative for serum HBsAg by currently available assays [2]. Actually, these patients represent the serologically detectable OBI fraction, because patients have been also described with detectable HBV DNA in the liver but undetectable HBV DNA in the serum. Accordingly, an international meeting stated OBI as "presence of HBV DNA in the liver (with detectable or undetectable HBV DNA in the serum) of individuals testing HBsAg negative by currently available assays" [2,3]. About one half OBI patients is positive for anti-HBc and/or anti-HBs (seropositive-OBI), while the other half is negative for both antibodies (seronegative-OBI) [2].

The mechanisms underlying OBI have been the object of several studies but remain to be clarified. Most published studies searching for HBV mutations possibly related to this particular clinical status focused on analysis of a fragment of HBV genome (mostly the region coding for HBsAg), while those studies analyzing complete HBV genomes focused on analysis of a specific HBV genotype from a small group of patients or from a specific geographic area [4-6]. No features common to all OBI genotypes have been identified.

At least in a fraction of OBI, absence of HBsAg might be due to mutations in HBsAg coding region, affecting export or leading to HBsAg with reduced immuno-reactivity with available monoclonal assays [7]. However, assay sensitivity cannot explain a major biological feature of OBI, i.e. the low level of serum HBV-DNA. The genome of HBV includes several regulative elements, such as promoters, Enhancer I and II which provide strong position- and orientation-independent increase of transcription from the major HBV promoters, the Post-transcriptional Regulative Element (PRE) promoting the export of viral transcripts otherwise inefficient due to absence of splicing, Direct Repeat 1 and 2 (DR1 and DR2) involved in genome replication, binding sites for transcription factors and others. Overall, these elements regulate, directly or indirectly, HBV replication, transcription and translation and, thus, any mutation affecting them might theoretically modify the precise modulation of HBV gene expression, essential for efficient replication of the virus. Whether or not mutations in regulative elements of HBV genome may be involved in OBI has not been clearly established.

In the present study, we attempted to search for point mutations possibly associated to OBI clinical status by analyzing complete genome HBV sequences belonging to several genotypes. A dataset of 41 complete genome HBV sequences from OBI patients ("OBI dataset"), and a dataset of 162 HBV sequences from non-OBI patients ("non-OBI dataset") were retrieved from GenBank. They were analysed by a computational approach including statistical evaluation. The feasibility of bioinformatics prediction models to classify HBV sequences into OBI and non-OBI by molecular data was also evaluated. In addition, we analyzed the predicted amino acid sequences of the S open reading frame (ORF) of OBI and non-OBI sequences for the presence of Stop codons, that might account for absence of serum HBsAg in OBI. Finally, high amino acid mutation rate in the Small S protein has been recently reported in genotype D OBIs [7]: we could evaluate S amino acid variability in genotype D only OBIs, due to the too small number of available complete genome sequences from other genotypes.

## Methods

### Datasets of HBV sequences

Two datasets of complete genome HBV sequences were built by downloading HBV sequences from GenBank: an "OBI dataset", i.e. a dataset of HBV sequences from OBI patients, and a "non-OBI dataset", i.e. a dataset of HBV sequences from patients with typical HBV infection.

OBI sequences were identified by database searching for "occult" and "hepatitis B" keywords. Each sequence was verified to be effectively classified by its authors as a true OBI sequence by examining annotations and/or by searching informations in the published study in which each sequence was reported. When more than one cloned sequence from the same patient was available, only one of them was randomly selected and included in the dataset. HBV sequences from patients reported to be infected by multiple (more than one) genotypes were not included. Overall, 41 OBI sequences from 41 patients could be retrieved, representing the A, C, D and E genotypes (Table 1).

**Table 1:** Summary of sequences included in OBI and non-OBI datasets, by country and genotype

Country	OBI dataset					non-OBI dataset				
	
	Total patients	HBV sequences by genotype				Total patients	HBV sequences by genotype			
				
		A	C	D	E		A	C	D	E
China	8		8			13		13		
Spain	2			2		1			1	
Ghana	9				9	9				9
France	1			1		3	2		1	
Italy	13	1		12		7			7	
India	8	4		4		7	4		3	
others						122	19	27	58	18
Total	41	5	8	19	9	162	25	40	70	27

The non-OBI control dataset was built by including sequences according to the following criteria: (a) matched non-OBI sequences described in the same studies reporting OBI sequences, (b) similar genotype representation as in the dataset of OBI sequences and (c) as much as possible, similar geographical origin as in the dataset of OBI sequences.

Overall, 162 non-OBI sequences were retrieved, representing the same A, C, D and E genotypes as in the OBI dataset. The ratio of OBI to wild-type sequences was roughly 1:4.

The complete list of Accession number of all sequences is reported in Additional File 1, Table S1.

### Univariable and multivariable sequence analysis

HBV sequences were aligned using Opal software [8]. Columns containing gaps and ambiguous base codes were treated as missing information, in order to be as much restrictive as possible with respect to putative sequencing errors.

The final alignment was encoded into a set of categorical variables for each base position and into another set of categorical variables for triplets of bases spanning the whole set of frame translations. The outcome variable was binary, coding OBI *vs*. non-OBI phenotype.

Univariable analysis was performed using Fisher's exact test on each categorical variable and the outcome, adjusting obtained p-values with Benjamini-Hochberg procedure [9].

Multivariable analysis was carried out either on the encoded input set of bases or triplets (whole set or reduced set by filtering positions found to be significant under univariable analysis) using either Logistic Regression (LR) with LogitBoost and AIC feature selection [10], or rule induction (RI) [11], or Random Forest (RF) feature evaluation with Gini index [12]. In order to account for the possible bias induced in RF by different and elevated number of categories, we performed an adjusted t-test statistic on the Gini distributions by comparing the values from 30 independent RF runs and 30 independent RF runs with outcome shuffling, similar to a previously presented procedure [13].

Extra-sample error estimation for LR, RI, and RF was assessed by Multiple ten-fold Cross-Validation (MCV), executed 10 times. Goodness-of-fit indicators were the Area Under the Receiver Operating Characteristic curve (AUC) [14], accuracy, specificity and sensitivity. Model comparison was made by executing t-test with Bengio's correction for sample overlap [15] on the AUC distributions obtained from the MCV runs for each model against the best-performing model.

Analyses were performed using R [16] and Weka open source software [17].

### Analysis of the Small S coding region of OBI and non-OBI sequences

The Small S of OBI and non-OBI nucleotide sequences was translated by the BioEdit Sequence Alignment Editor [18] and the obtained amino acid sequences were analyzed for the presence of Stop codons.

To compare variability of genotype D OBI and non-OBI sequences, entropy values (Hx) along the 226 amino acids of the Small S protein were calculated and plotted using the Entropy plot tool of BioEdit. The entropy value is a measure for nucleotide or amino acid variation at a given position in aligned sequences; it varies from 0 (i.e. no variation) to 3.04 (i.e. all the possible 20 amino acids or a gap occur in equal frequency).

The Major Hydrophilic Region (MHR) of 19 genotype D OBI amino acid sequences was analyzed for substitutions by comparison with a consensus MHR from 70 genotype D non-OBI sequences, that resulted to be identical to the consensus obtained by Chaar et al. from 20 genotype D non-OBI sequences [7].

## Results

### Univariable analysis

Sequences from the OBI and non-OBI datasets were compared by univariable analysis. Table 2 summarizes HBV nucleotide positions (categorical variables) significantly enriched in the OBI dataset *vs*. non-OBI dataset, as well as the corresponding amino acid changes.

**Table 2:** Nucleotide positions significantly enriched in OBI dataset by univariable analysis

Nucleotide positions*	Adjusted p-value	Significant nt changes in OBI *vs*. non-OBI	Amino acid change in affected *open reading frames*				Affected HBV protein**
				
			polymerase	surface		core	
						
				pre-S2	S		
78	0.0144	G → A	no change	Gly → Glu			L, M
233	0.0367	A → G	His → Arg (RT domain)		Thr → Ala		P, L, M, S
418	0.0367	G → T	Ala → Ser (RT domain)		no change		P
2240 ***	0.0097	A → G				Thr → Val (T helper epitope) ***	C
2241 ***	0.0144	C → T				Thr → Val (T helper epitope) ***	C
2435	0.0367	C → A	no change			Gln → Lys (Arg-rich C-terminus)	C
2485	0.0367	A → G	Asn → Ser (TP domain)				P

* positions in the well-annotated reference HBV sequence Acc.No. AM282986

** L, M, S: Large, Middle and Small S, respectively; P: Polymerase; C: Core

*** nucleotides 2240 and 2241 co-variated in all observed cases, substituting ACT with GTT or ACA with GTA codons.

Significant nucleotides are distributed in three ORFs: polymerase (in the RT and TP domains) [19], surface (in the Pre-S2 and S domains) and core (T-helper epitope region and Arg-rich C-terminus) [20,21]; none affects the X ORF.

Due to the organization of HBV genome with overlapping coding regions, four positions (nucleotide 78, 233, 418 and 2435) affect simultaneously two ORFs. However, only one of them (nucleotide 233) actually leads to amino acid change in both frames; by virtue of code degeneration, the remaining three positions (78, 418 and 2435) produce no amino acid change in one of the two overlapping frames, because they occur in third position of the affected codon. Actually, at the protein level the nucleotide change in position 78 affects two proteins, the Large and Middle S, because it is located in the Pre-S2 region, shared by these proteins. Position 233 has even higher impact; besides producing amino acid change in two ORFs, it affects four proteins: in addition to the Polymerase, the Large, Middle and Small Surface proteins are affected, because position 233 is located in the region shared by these three proteins (Table 2).

Careful examination of the well-annotated reference HBV sequence Acc. No. AM282986 showed that, according to annotations herein reported, none of the identified significant positions occurs in known regulative regions of HBV genome, such as promoters, enhancer I and II, Post-transcriptional Regulative Element (PRE), binding sites for cellular transcription factors, *et cetera*; likewise, none of the significant positions occurs in untranslated regions of HBV mRNAs. Thus, any possible contribution of identified nucleotide variants to the OBI "phenotype" appears to be due to the amino acid change in the affected proteins.

Overall, 11 out of 41 OBI sequences (26.8%) showed significant changes. Thus, the observed variations might be involved in a fraction but not most OBI.

### Multivariable analysis

Multivariable Logistic Regression (LR) with backward Akaike information criterion (AIC) stepwise selection was executed on the set of bases/triplets significantly associated with OBI and non-OBI groups under the univariable Fisher's test, in order to assess their importance by mutual adjustment. Additionally, by considering the full set of bases/triplets, and 30 independent runs of Random Forest (RF) models, the average Gini index was used as a measure of variable importance. In addition, Decision Tree (DT) and Rule Induction (RI) models were trained on the full data sets. All models were analysed in terms of different goodness-of-fit functions, estimating the extra-sample error via Multiple ten-fold Cross-Validation (MCV).

**Figure 1:**
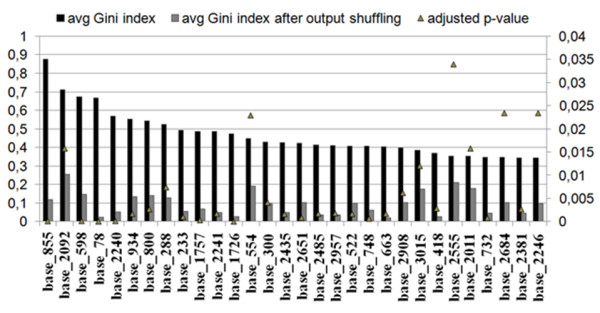
**Average Gini index as a measure of variable importance**. Averaged Gini variable importance extracted from 30 random forest models trained on the full data set, compared with the average Gini values obtained by re-shuffling the class variable. Variables are ranked decreasingly by their importance. Only variables with a p-value < 0.05 are depicted.

Whilst the LR did not produce any set of predictors showing independent association with the outcome, the analysis of the Gini importance from the RF yielded several significant positions, and all those found to be significant by the univariable analysis were confirmed (see Figure 1 and Table 2).

### Performance of the models

Performance of different classification models in terms of Area Under the receiver operating Characteristic (AUC), accuracy, true negative rate, and true positive rate was evaluated. 10 independent runs of 10-fold cross-validation were executed, using different input sets and feature selection methods.

The best model in terms of AUC and accuracy was a RF with the whole set of bases or triplets as input set (Table 3). The RF trained with the bases as input encoding yielded an average AUC of 0.847 (st.dev.: 0.100) and accuracy of 83.948 (st.dev.: 5.024).

**Table 3:** Performance of different classification models

input encoding	model	feature selection	average AUC (st. dev.)	average accuracy (st. dev.)	average TNR (st. dev.)	average TPR (st. dev.)
bases	**RF**	none	0.847 (0.100)	83.948 (5.024)	0.220 (0.230)	0.992 (0.021)
triplet	**RF**	none	0.847 (0.097)	83.952 (4.820)	0.224 (0.222)	0.990 (0.022)
bases	**RF**	Fisher	0.699 (0.160) *	81.462 (5.812)	0.215 (0.226)	0.962 (0.055)
triplet	**RF**	Fisher	0.759 (0.127)	81.781 (5.306)	0.234 (0.219)	0.961 (0.047) *
bases	**LR**	Fisher/AIC	0.670 (0.134) *	81.310 (6.036)	0.283 (0.220)	0.943 (0.054) *
triplet	**LR**	Fisher/AIC	0.680 (0.147) *	81.343 (6.878)	0.324 (0.226)	0.934 (0.058) *
bases	**DT**	none	0.570 (0.137) *	79.862 (5.904)	0.143 (0.217)	0.960 (0.059)
triplet	**DT**	none	0.549 (0.106) *	80.136 (4.850) *	0.130 (0.203)	0.967 (0.059)
bases	**RI**	none	0.574 (0.094) *	80.662 (5.054)	0.190 (0.219)	0.958 (0.072)
triplet	**RI**	none	0.579 (0.110) *	79.943 (5.442)	0.215 (0.249)	0.943 (0.074)

* worse than RF on whole set of bases at p < 0.05

AUC: area under the receiver operating characteristic; RF: random forest; LR: logistic regression; DT: decision tree; RI: rule induction; TNR: true negative rate; TPR: true positive rate; AIC: Akaike information criterion.

With respect to the other models, i.e. LR, RI and DT, RF was always superior in terms of AUC and accuracy, but not always when considering the true positive or true negative rate. A reduction of the input set based on the inclusion of positions significant under univariable analysis did not produce any improvement in the model fits.

**Figure 2:**
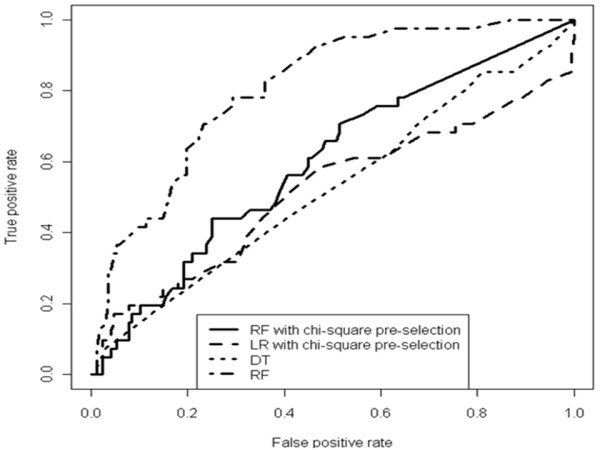
**True positive *vs*. false positive rate plots of different models trained on the full data set**. A higher "Area Under the Curve" (AUC) means a higher chance to classify a random isolate from the occult group as a true occult hepatitis, as compared to the chance to classify a random isolate from the wild type group as an occult hepatitis. RF: Random Forest; LR: Logistic Regression; DT: Decision Tree.

Of note, average specificity (true negative rate) was low for all models, although at a fixed false positive rate (1-specificity) of 10%, the best RF model was able to give a 56% of sensitivity (Figure 2).

### Analysis of Surface ORF for presence of Stop codons

Stop codons in the Surface ORF might theoretically account for the absence of serum HBsAg in OBI patients. Thus, the region coding for the Small surface protein from OBI and non-OBI cases of our dataset was analyzed for the presence of Stop codons. The frequency was found to be higher in OBI than non-OBI sequences [3 out of 41 (7.3%) OBI *vs*. 5 out of 162 (3.1%) non-OBI sequences], but the difference did not reach statistical significance (Fisher's exact test: p = 0.19).

### Analysis of genotype D OBI sequences for variability in the Small S protein

Recently, high substitution rate in the Small S protein of genotype D OBI strains, in particular in the Major Hydrophilic Region (MHR), has been reported; substitution rate in the MHR was found to be higher in anti-HBs positive than in anti-HBs negative OBIs, suggesting an escape mutation mechanism related to imperfect immune control, that would result in low level viral replication [7].

**Figure 3:**
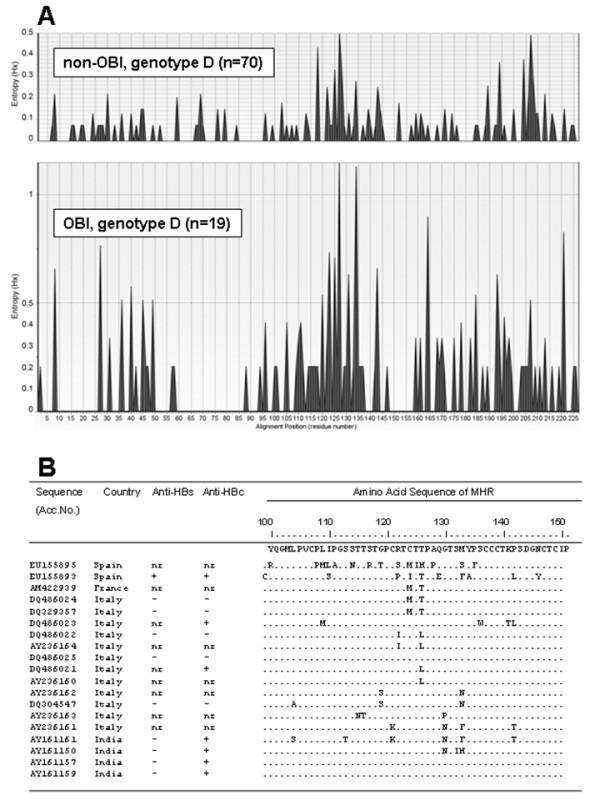
**Variation of the Small S protein of genotype D OBI and non-OBI sequences**. **A**: variation along the complete Small S protein of genotype D OBI and non-OBI sequences shown by entropy plots (see Materials and Methods). Entropy values (Hx) are a measure of variation at each amino acid position in the set of aligned sequences. It can vary from 0 (i.e. no variation) to 3.04 (i.e. all the possible 20 amino acids or a gap occur in equal frequency in a position. **B**: amino acid variations in the MHR of HBsAg in 19 genotype D OBI sequences *vs*. a consensus from genotype D non-OBI sequences. Accession number and country of origin of each sequence are reported.

We evaluated variation along the 226 predicted amino acids of the Small S protein of genotype D OBI and non-OBI sequences by calculating entropy values (Hx). Comparison of the resulting entropy plots showed higher entropy values in OBI than in non-OBI group, despite the higher number of non-OBI sequences (Figure 3A). The most prominent regions of amino acid variation correspond to amino acids of the "a" determinant of the S protein (residues 110-140), located inside the MHR region. These findings confirm that variability in Small S protein in genotype D is higher in OBIs than in non-OBIs. The observed amino acid substitutions in the MHR of the 19 genotype D OBI sequences, as resulting by comparison with a consensus from non-OBI sequences, are reported in Figure 3B. Unfortunately, we could not compare anti-HBs positive *vs*. anti-HBs negative OBIs, because most complete genome sequences for which patient anti-HBc/anti-HBs status could be retrieved were anti-HBs negative.

## Discussion

The most prominent features of occult hepatitis B are absence of detectable HBsAg and low level viremia. Although the underlying mechanisms involved in OBI remain to be clarified, both features might be due to low level HBV replication and expression, and it can be hypothesized that at least in some cases they might be caused by point mutations in regulative elements implicated in the control of viral replication and expression. Actually, data from the present study do not support this hypothesis: no nucleotide variations found to be significantly enriched in the OBI dataset mapped to known viral regulatory regions. Thus, the only possible contribution of such variations to OBI appears to be by amino acid change of HBV proteins.

Mechanisms potentially able to explain low level viremia might involve steps of the replicative cycle such as assembly, budding and entry of viral particles and/or the efficiency of viral particle removal by immune system; indeed, a low viral load might also result from low level replication in the presence of incomplete immune control. Mutation in viral proteins might affect any of the above processes: however, the possible mechanistic biological link with the occult state is not obvious for all identified significant amino acid changes.

The identified G to A change of nucleotide 78 produces a Gly to Glu amino acid change in the Pre-S2 region of the Surface ORF, thus replacing a neutral residue with a negatively charged one. Both the Large and the Middle S proteins include the amino acids encoded by the Pre-S2 region, thus both proteins are affected by the observed change. The Large S, due to alternative folding, exists in the form of two alternative structures, one of them mediating virion assembly and the other involved in virion binding to the cellular receptor. According to the current model of Large S protein structure, it can be speculated that the Gly to Glu change in the Pre-S2 region might affect protein folding and, thus, one or both processes.

The 233, 418 and 2485 nucleotide substitutions produce amino acid change in the polymerase, and they might be directly involved in the replication efficiency of HBV genome by affecting polymerase activity. In addition, the 233 substitution also affects the Small S ORF, shared by the Large S, Middle S and Small S proteins, producing a Thr (hydrophilic) to Ala (hydrophobic) amino acid change. Thus, the 233 substitution impacts simultaneously on 4 different HBV proteins.

Finally, the AC to GT co-variation of nucleotides 2240 and 2241, and the C to A change of nucleotide 2435 resulted in amino acid change in the core ORF, the first one affecting a T-helper epitope and thus, possibly interfering with immune response, the other replacing a polar uncharged with a positively charged amino acid, in a region already rich of positively charged amino acids.

On the whole, results show that the Polymerase, the Large, Middle and Small S and the Core proteins, but not the X, are the targets of the observed significant variations. These findings imply several different viral proteins may contribute to OBI and suggest that heterogeneous mechanisms are involved in the genesis or maintenance of occult clinical status.

Overall, in our dataset only 11 out of 41 (26.8%) OBI sequences showed the identified significant amino acid variants. Likely, this result might be due to the small size of the OBI dataset, which affects statistical significance and might have precluded identification of further relevant positions. Nevertheless, it cannot be excluded that point mutations of HBV genome effectively play a significant role in one third only OBIs, with other factors being involved in the remaining two thirds. Among other possible factors might be: (a) co-infection with other viruses, such as Hepatitis C Virus (HCV), that might interfere with HBV replication: indeed OBI has been frequently identified in HCV infected patients [reviewed in 22]; (b) incomplete immune control by the host; (c) DNA methylation of HBV genome [23]; (d) large deletions, rather than point mutations, in regulative elements of HBV genome, as reported in genotype C OBI patients [24].

Due to the small size of the OBI dataset, we could not carry out intra-genotype univariable and multivariable analysis; thus, our data cannot exclude also the existence of genotype specific variations associated with OBI.

Although the frequency of Stop codons in the S ORF was higher in OBI than non-OBI sequences, the difference was not statistically significant. It appears that absence of circulating HBsAg due to mutations introducing a Stop codon in the Small S is not a general mechanism responsible for OBIs, though could be involved in a fraction of them. Further studies with larger datasets are needed to better address this point.

An escape mutation mechanism has been associated with genotype D OBI on the basis of a high substitution rate in the S protein, particularly in the MHR of anti-HBs positive OBIs [7]. Analysis of entropy plots of genotype D sequences from our OBI and non-OBI datasets showed higher variability in OBIs (Figure 3A): the most prominent regions of amino acid variation correspond to amino acids 110-140, a region spanning the "a" determinant of the HBV S protein. The "a" determinant is located within the immunodominant loops and is included in the MHR (residues 100-151). It is exposed on the surface of HBV particle and represents a highly immunogenic region, it is the primary target of neutralizing antibodies and it is common to all HBV genotypes. However, it remains to be investigated whether in genotype D OBIs such an escape mechanism is the main factor or one of several factors responsible for OBI, as well as whether or not this mechanism also plays a role in the other genotypes.

Prediction models are being developed as promising tools to help clinician in diagnosis and patient management. In the present study, we evaluated the feasibility of bioinformatics prediction models to classify HBV infections into OBI and non-OBI by molecular data. The performance of the models was evaluated by accuracy, AUC, TNR and TPR, four parameters measuring the prediction property of the test. The overall prediction performance, although showed high accuracy and AUC, may not be satisfactory in terms of specificity (TNR and TPR), for any in-silico genotype-based phenotype prediction model (Table 3). A more complex input encoding, considering base triplets instead of single base positions, produced similar results. The unsatisfactory performance was likely due to the relatively too small sample size of OBIs and the highly unbalanced (4:1) dataset towards non-OBI. However, results are promising in the perspective of a broader collection of OBI sequences and indicate the feasibility to derive prediction models to classify HBV infections into OBI and non-OBI by molecular data.

## Conclusions

In summary, computational analysis of complete genome HBV sequences from OBI and non-OBI patients showed several different point mutations significantly associated with OBI. All of them were involved in amino acid change and none mapped to regulative elements of HBV genome. All viral proteins, with the only exception of the X, were found to be targets of the significant variations, suggesting heterogeneous mechanisms may contribute to OBI. It is likely that further variations associated with OBI did not achieve statistical significance due to the too small size of available OBI dataset. This factor also limited the specificity of prediction models, whose performance might improve in the near future when a larger set of complete genome OBI sequences will become available as a result of the newly introduced "massively parallel sequencing" techniques.

## List of abbreviations used

AIC: Akaike information criterion; AUC: Area Under the receiver operating Characteristic; DT: decision tree; HBV: hepatitis B virus; L: large surface protein; LR: logistic regression; M: middle surface protein; MHR: Major Hydrophilic Region; OBI: occult B infection; RF: random forest; RI: rule induction; S: small surface protein; TNR: true negative rate; TPR: true positive rate.

## Competing interests

The authors declare that they have no competing interests.

## Authors' contributions

RB and ARC have been involved in conception and design of the study, analysis and interpretation of data and in drafting and revising the manuscript critically; MP has been involved in design of the study, univariable and multivariable analysis, and in drafting the manuscript; MC participated in design of the study; CM and AA have been involved in data analysis and building of sequence datasets; UV, ET and ALP participated in data analysis. All authors read and approved the final manuscript.

## Supplementary Material

Additional file 1 **Accession Number of sequences from non-OBI and OBI patients**. Accession Number list of the sequences from non-OBI and OBI patients, downloaded from public databases and analysed in the present study. Click here for file
